# Lenvatinib Beyond First-Line Therapy in Patients With Advanced Biliary Tract Carcinoma

**DOI:** 10.3389/fonc.2022.785535

**Published:** 2022-03-03

**Authors:** Yunchao Wang, Xiaobo Yang, Dongxu Wang, Xu Yang, Yanyu Wang, Junyu Long, Jinxue Zhou, Zhenhui Lu, Yilei Mao, Xinting Sang, Mei Guan, Haitao Zhao

**Affiliations:** ^1^ Department of Liver Surgery, Peking Union Medical College Hospital, Chinese Academy of Medical Sciences and Peking Union Medical College, Beijing, China; ^2^ Department of Hepatobiliary Surgery, General Surgery, Qilu Hospital, Cheeloo College of Medicine, Shandong University, Jinan, China; ^3^ Department of Hepatopancreatobiliary Surgery, The Affiliated Tumor Hospital of Zhengzhou University, Zhengzhou, China; ^4^ Hepatobiliary and Pancreatic Surgery, Shenzhen Qianhai Shekou Free Trade Zone Hospital, Shenzhen, China; ^5^ Department of Oncology, Peking Union Medical College Hospital, Chinese Academy of Medical Sciences, and Peking Union Medical College, Beijing, China

**Keywords:** lenvatinib, advanced biliary tract carcinoma, efficacy, biomarker, tyrosine kinase inhibitor

## Abstract

**Introduction:**

Lenvatinib, a multiple receptor tyrosine kinase inhibitors that target vascular endothelial growth factor receptors and fibroblast growth factor receptors, recently demonstrated a treatment effect in various tumors. This study evaluated the efficacy and safety of lenvatinib for patients with biliary tract cancers (BTCs) who had received ≥1 line of prior systemic anti-BTC therapy.

**Methods:**

This open-label, single-arm study included adult (≥18 years) patients with histologically confirmed BTC. Efficacy and safety were evaluated based on the Response Evaluation Criteria in Solid Tumors RECIST Version 1.1 (RECIST 1.1) and the National Cancer Institute Common Terminology Criteria for Adverse Events (CTCAE version 4.0). Changes in tumor biomarkers throughout the treatment period were recorded.

**Results:**

41 patients received lenvatinib treatment. The ORR was 12% (95% CI: 1.7–22.7), with a median PFS of 3.8 months (95% CI: 1.3–6.3) and an OS of 11.4 months (95% CI: 6.6–16.2). Thirty-nine (95.1%) patients experienced ≥1 treatment-related adverse event. Decreasing carbohydrate antigen 19-9 (CA19-9) level predicted tumor size reduction in intrahepatic cholangiocarcinoma with a sensitivity of 77.7% and a specificity of 73.9%.

**Conclusions:**

Lenvatinib which was individualized based on the patient’s weight has promising clinical activity against advanced BTC and had an acceptable safety profile. Additionally, serum biomarkers and gene sequencing may hold the potential to guide our treatment.

## Introduction

Biliary tract cancers (BTCs), encompassing intrahepatic cholangiocarcinoma (ICC), extrahepatic cholangiocarcinoma (ECC), and gallbladder cancer (GBC), are aggressive neoplasms with a dismal prognosis (1). The global incidence of BTCs has increased in recent years, in large part due to an increase in the incidence of ICC (2). Surgical resection is regarded as a curative treatment option for patients with early-stage BTC. However, most patients present with unresectable or metastatic disease at diagnosis. Presently, gemcitabine combined with cisplatin (GC) chemotherapy is the standard first-line treatment for advanced BTCs (3–5), and the incidence of adverse reactions accounted for 70%. The ABC-06 study evaluated the benefit derived from FOLFOX (folinic acid, fluorouracil, and oxaliplatin) chemotherapy in patients with advanced BTC and the median overall survival (OS) was improved significantly compared to active symptom control (ASC) (6.2 months *vs* 5.3 months). However, the absolute benefit was not large, and the median OS was only prolonged by 0.9 months (6). At the same time, the incidence of the grade 3-5 AEs associated with FOLFOX chemotherapy account for 69%. For patients who progress on or after the above therapies, the subsequent treatment options are limited.

Multiple molecular pathways, including those mediated by vascular endothelial growth factor (VEGF) and fibroblast growth factor receptor (FGFR), are crucial in the carcinogenesis and development of BTC. In particular, overexpression of VEGF occurs in 42% to 76% of patients with advanced unresectable BTC and is associated with a poor prognosis (7). Multiple small studies of treatments targeting the VEGF pathway (sorafenib, cabozantinib, bevacizumab, sunitinib, or axitinib) have been conducted in BTC and showed modest efficacy (8–11). Furthermore, FGFR plays an important role in regulating cell proliferation, migration, invasion, and angiogenesis in BTC. Previous studies have found FGFR fusions in patients with BTC and shown that the presence of these alterations predicts a more favorable prognosis (7). Several clinical trials of drugs that target FGFR-fusions have been conducted, including infigratinib, pemigatinib, and derazantinib (12).

Lenvatinib is a small-molecule tyrosine kinase inhibitor of VEGFR1-3, FGFR1-4, platelet-derived growth factor receptor a (PDGFRa), stem cell factor receptor (KIT), and rearranged during transfection (RET). Lenvatinib promotes tumor shrinkage in hepatocellular carcinoma (13), endometrial carcinoma (14, 15), and renal cell cancer (16). In a previous study, we found lenvatinib plus pembrolizumab was a potentially effective and tolerable non-first-line treatment for refractory BTC, with an objective response rate (ORR) of 25%, a clinical benefit rate (CBR) of 40.5%, a median progression-free survival (PFS) of 4.9 months, and a median OS of 11.0 months (17). As lenvatinib can inhibit multiple kinases that are implicated in BTC carcinogenesis, and due to the positive effects observed when lenvatinib is used as a combination therapy, we sought to test lenvatinib monotherapy, as this may provide a better safety profile while remaining efficacious. In the present study, we investigated the efficacy and safety of lenvatinib monotherapy in patients with advanced BTC.

## Materials and Methods

### Study Design

This was an open-label, single-arm study designed to assess the efficacy and safety of lenvatinib as a non-first-line treatment in patients with advanced BTC. Clinical data from patients with advanced BTC treated with lenvatinib in second-line or beyond were collected from December 2017 until July 2019. All patients had been diagnosed with unresectable BTC and were routinely attending for a treatment consultation. The study protocol was compliant with the Declaration of Helsinki and was approved by the Institutional Review Board and Ethics Committee at Peking Union Medical College Hospital. All patients provided written, informed consent before enrolment. The study protocol was prospectively registered at ClinicalTrials.gov (NCT04656249).

### Patients

Eligible patients were aged ≥18 years with histologically confirmed ECC, ICC, or GBC and had previously received at least one systemic anti-BTC therapy. Other eligibility criteria included at least one measurable or evaluable tumor lesion according to Response Evaluation Criteria in Solid Tumors version 1.1 (RECIST 1.1) and an Eastern Cooperative Oncology Group performance status of 0–2. Participants who received adjuvant chemotherapy were eligible if this therapy was completed and no recurrence was observed for 6 months after completion of the therapy. Key exclusion criteria included intolerance to lenvatinib, the life expectancy of ≤3 months, moderate or severe ascites or ascites requiring drainage, proteinuria of ≥2+ on dipstick testing, gastrointestinal malabsorption, or any other condition that the investigator determined may affect the absorption of study drug, cardiovascular disease, HIV infection, and gastrointestinal bleeding.

### Treatment

Patients with a bodyweight <60 kg received 8 mg of lenvatinib and those with a bodyweight ≥60 kg received 12 mg orally once a day in 28-day cycles until disease progression or intolerable toxicity occurred. Dose interruption and reduction of lenvatinib (to 10mg QD) were permitted for drug-related adverse events. Lenvatinib could be interrupted and even discontinued with the occurrence of unacceptable or serious adverse events (AEs) or due to disease progression.

### Assessments

We evaluated tumor size using enhanced computed tomography (CT), magnetic resonance imaging (MRI), or other available imaging examinations according to RECIST 1.1 at 4–8 weeks after the patient-initiated lenvatinib at the stipulated dose. Imaging examination was conducted by a specialist to assess tumor size changes and each patient was categorized as either complete response (CR), partial response (PR), stable disease (SD), or progressive disease (PD). Disease control rate (DCR; CR+PR+SD) and ORR (CR+PR) were also calculated.

The therapeutic efficacy included ORR, PFS, OS, DCR, and safety. Treatment-related AEs were categorized and graded according to the National Cancer Institute Common Terminology Criteria for Adverse Events, version 4.0 (CTCAE 4.0). The highest grade for each AE experienced by each patient during the observation period was recorded. Treatment of AE symptoms was incorporated into treatment regimens for patients experiencing grade 1 or 2 AEs. IF grade 3 AEs occurred, the treatment dose was reduced or treatment was temporarily interrupted until symptoms were reduced to grade 1 or 2. If grade 4 AEs occurred, the administration of lenvatinib was discontinued.

### Tumor Serum Biomarker and Gene Sequence Data Analysis

Serum concentrations of carbohydrate antigen 19-9 (CA19-9) were recorded every 6-8 weeks from lenvatinib initiation until treatment discontinuation or completion of the study period. We assessed the ability of biomarker values before and after treatment to predict the direction and amount of change in tumor size by receiver operating characteristic (ROC) curve analysis. In addition, patients with BTC were divided into the following groups: partial response (PR), diminished tumor size that did not reach the partial response standard (SS), stable disease without any significant tumor size change (ST), and progressive disease (PD). Gene mutation information was collected from the patients by performing gene sequencing of tumor tissue. For each group, different information representing the gene mutation was calculated.

### Statistical Analysis

PFS and OS were calculated from the initiation of lenvatinib until disease progression or BTC-related death. The clinical baseline data of patients including continuous variables and categorical variables were summarized as averages with corresponding standard deviations or simple numbers and percentages according to their types. The Kaplan-Meier method was used to generate PFS and OS curves. The sensitivity and specificity of diagnostics were calculated to assess their predictive capabilities for tumor changes using CA19-9 values. Gene mutation frequencies were used to construct a gene mutation map of patients with different therapeutic outcomes. SPSS software system (vision 22.0, SPSS, Inc., Chicago, IL), and GraphPad Software (vision 8.0, GraphPad Software, San Diego, California) were used for statistical analysis.

## Results

### Patient Demographics and Baseline Characteristics

**Figure 1:**
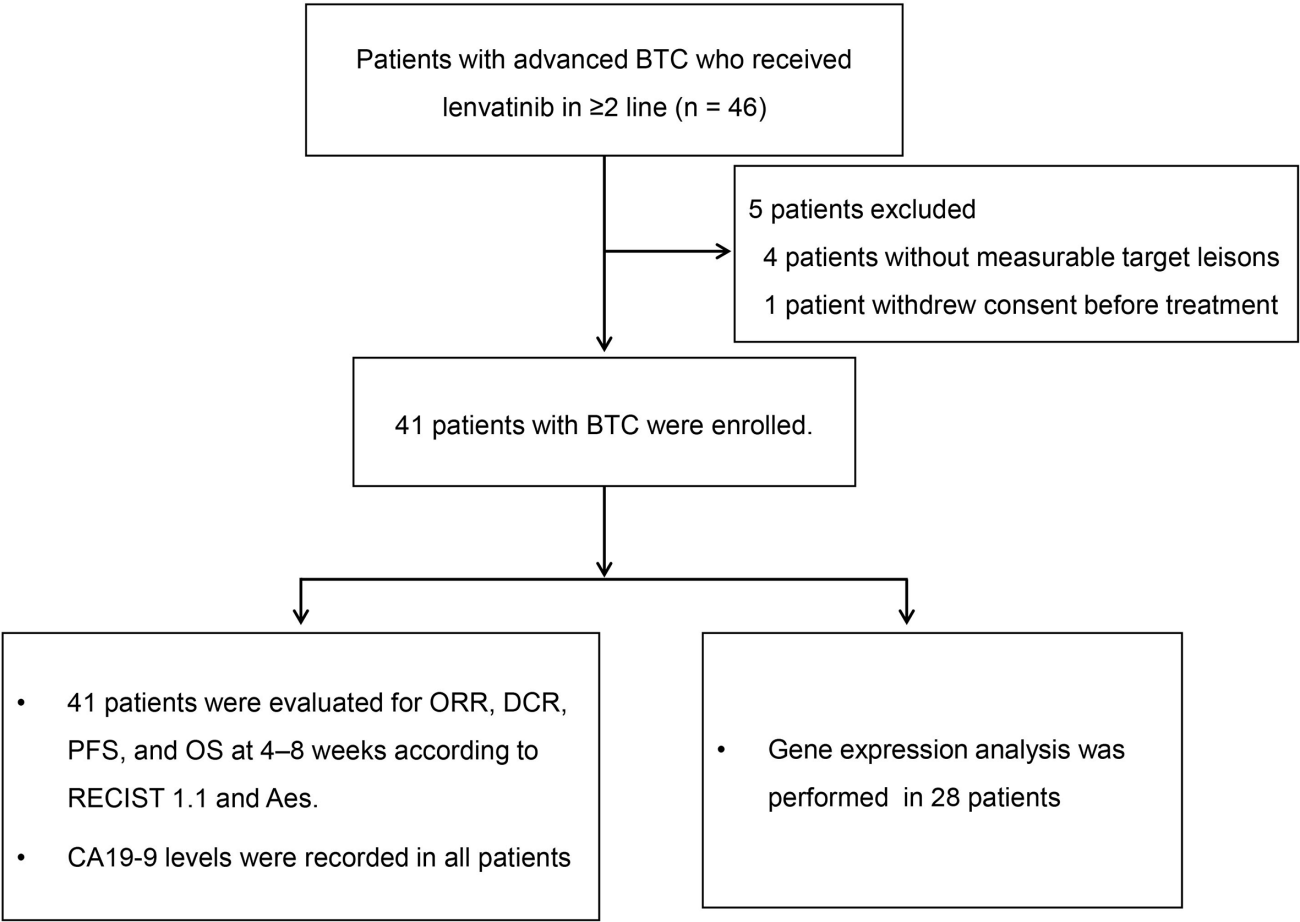
Study flow diagram. BTC, biliary tract cancer; CA19-9, carbohydrate antigen 19-9; DCR, disease control rate; ORR, objective response rate; OS, overall survival; PFS, progression-free survival; RECIST 1.1, Response Evaluation Criteria in Solid Tumors version 1.1.

From December 2017 until July 2019, a total of 46 patients were enrolled. Of these patients, five were excluded: 4 patients without measurable target lesions, 1 patient withdrew consent before treatment (**Figure 1**). The mean age was 61 ± 12 years, and 61% were male. In total, 34 patients (82.9%) had ICC, three (7.3%) had ECC, and four (9.8%) had GBC. There were six patients with a hepatitis B infection. Twenty-three patients had received systemic chemotherapy (primarily gemcitabine with either cisplatin or oxaliplatin). Twenty-one patients (51.2%) had received more than one line of systemic treatment before initiating lenvatinib. Five patients (12.2%) had also received radiation therapy, and 11 patients (26.8%) had undergone transhepatic arterial chemoembolization. Seven patients (17.1%) had received radiofrequency ablation. Baseline characteristics of the patients included in this study are shown in **Table 1**.

**Table 1:** Patient demographics and baseline characteristics.

Characteristics	n = 41
Age, years	61.10 ± 12.50
Gender, n (%)	
Male	25 (61.0)
Female	16 (39.0)
Tumor subtype, n (%)	
ECC	3 (7.3)
ICC	34 (82.9)
GBC	4 (9.8)
ECOG performance status, n (%)	
0	12 (29.3)
1	25 (61.0)
2	4 (9.8)
HBV infection	6 (15.6)
Differentiated histology, n (%)	
Well	1 (2.4)
Moderately	8 (19.5)
Poorly	3 (7.3)
Moderately-poorly	7 (17.1)
Unsure	22 (53.7)
Total bilirubin, mg/dL	21.33 ± 22.43
Albumin, g/dL	37.77 ± 8.51
Site of Metastases n, (%)	
Intrahepatic	29 (70.7)
Lymph nodes	30 (73.2)
Bone	1 (2.4)
Lung	1 (2.4)
Other	2 (4.9)
Extent of disease, n(%)	
Native	21 (51.2)
Recurrence	20 (48.8)
Previous treatment regimens, n (%)	
Radical surgery resection	19 (46.3)
Systemic chemotherapy	23 (56.1)
TACE	11 (26.8)
RFA	7 (17.1)
Radiotherapy	5 (12.2)
≥ 2 lines of previous systemic treatments, n (%)	21 (51.3)
Initial dose of lenvatinib, n (%)	
8 mg	21 (51.2)
>8 mg	20 (40.8)
Hepatocirrhosis, n (%)	3 (7.3)
Gastric varices and portal hypertension, n (%)	3 (7.3)

ECC, extrahepatic cholangiocarcinoma; ICC, intrahepatic cholangiocarcinoma; GBC, gallbladder cancer; HBV, hepatitis B virus; HCV, hepatitis C virus; ICC, intrahepatic cholangiocarcinoma; RFA, radiofrequency ablation; TACE, transhepatic artery chemoembolization.

### Efficacy and Safety

**Figure 2:**
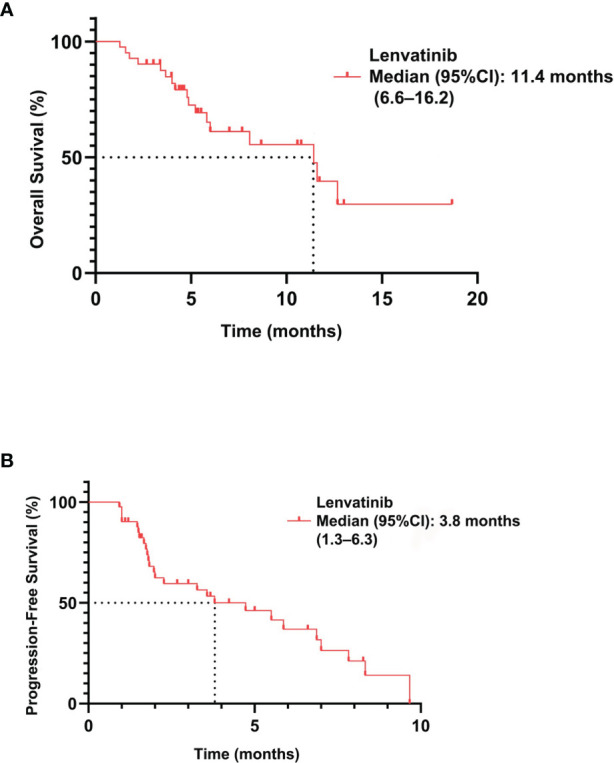
Overall survival **(A)** and progression-free survival **(B)** in patients with biliary tract carcinoma treated with lenvatinib (n = 41).

**Figure 3:**
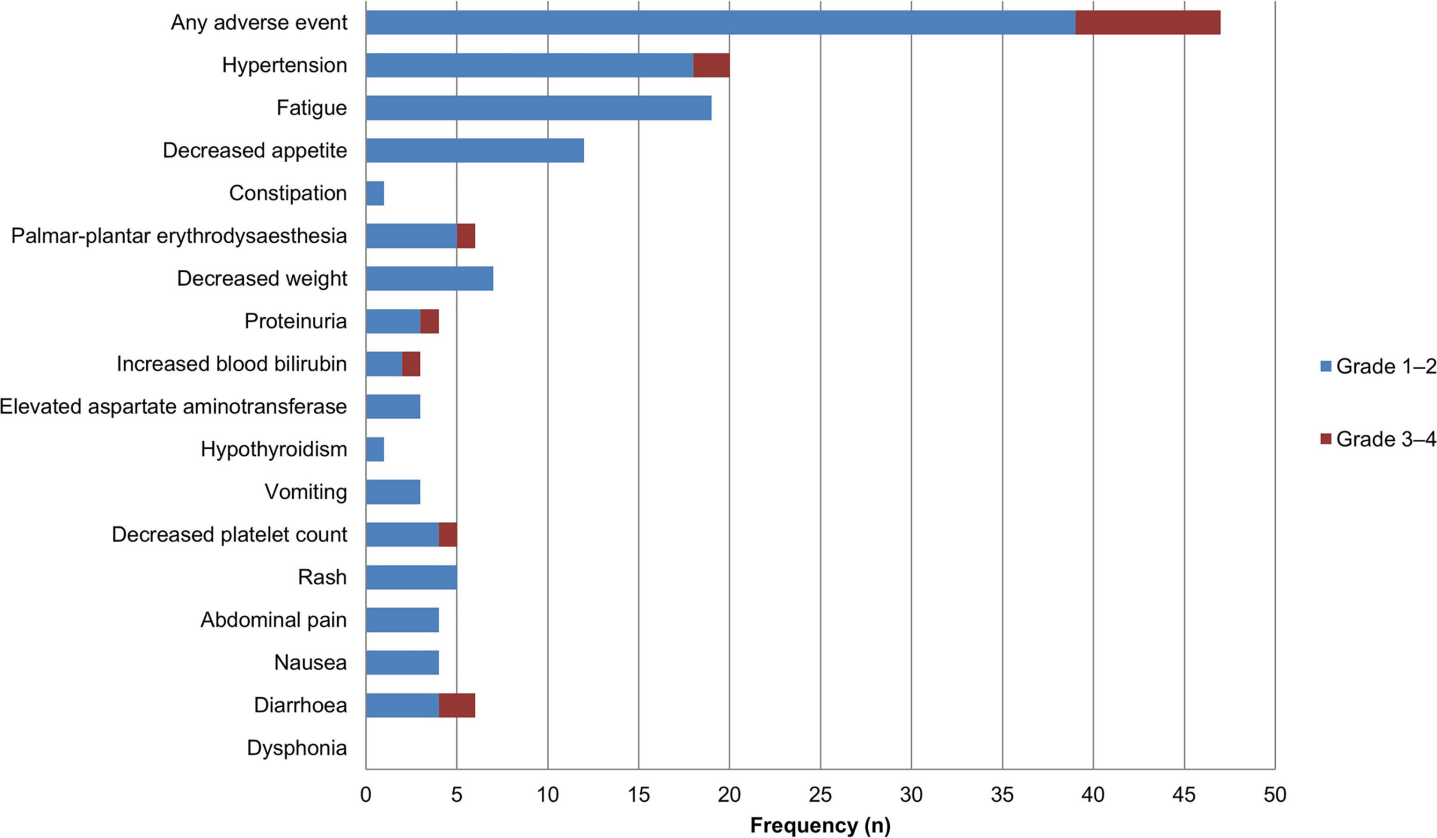
Adverse events during lenvatinib treatment in patients with biliary tract carcinoma (n = 41). Grade 1–2 adverse events are represented in blue, and grade 3–4 adverse events are represented in red.

Among 41 patients evaluable for tumor response, none achieved a CR, five achieved a PR, 27 had SD, and nine had PD (**Table 2**). The ORR was 12.2% (5/41; 95% CI: 1.7–22.7) and the DCR was 78.0% (32/41; 95% CI: 64.8–91.3). The median PFS was 3.8 months (95% CI: 1.3–6.3) and the OS was 11.4 months (95% CI: 6.6–16.2) (**Figure 2**). A total of 39 patients (95.1%) experienced ≥1 treatment-related AE, the most common of which were fatigue (46.3%, n = 19) followed by hypertension (43.9%, n = 18). Eighteen patients (19.5%) experienced grade 3–4 AEs, of which diarrhea and hypertension were the most common (both n = 2, 4.9%). A detailed list of AEs and their associated frequencies are shown in **Table 3** and **Figure 3**.

**Table 2:** Tumor responses based on RECIST 1.1 and predictive ability of biomarkers.

Response by investigator review (RECIST 1.1)	N = 41
Median overall survival, months (95% CI)	11.4 (6.6–16.2)
Median progression-free survival, months (95% CI)	3.8 (1.3–6.3)
Objective response rate (n, %), 95% CI	5 (12.2), 1.7–22.7
Complete response (n, %)	0 (0)
Partial response (n, %)	5 (12.2)
Stable disease (n, %)	27 (65.9)
Progressive disease (n, %)	9 (22.0)
DCR (n, %), 95% CI	32 (78.0), 64.8–91.3
Decreased CA19-9 predict DCR status	
Sensitivity, %	59.0
Specificity, %	88.8

CA19-9, carbohydrate antigen 19-9; DCR, disease control rate; RECIST, Response Evaluation Criteria in Solid Tumors.

**Table 3:** Summary of adverse events.

Events, n (%)	Biliary tract carcinoma (N = 41)	
	Any grade	Grade 3–4
Hypertension	18 (43.9)	2 (4.9)
Fatigue	19 (46.3)	0 (0)
Decreased appetite	12 (29.3)	0 (0)
Diarrhoea	4 (9.6)	2 (4.9)
Proteinuria	3 (7.3)	1 (2.4)
Decreased weight	7 (17.1)	0 (0)
Palmar-plantar erythrodysaesthesia	5 (12.2)	1 (2.4)
Nausea	4 (9.6)	0 (0)
Abdominal pain	4 (9.6)	0 (0)
Rash	5 (12.2)	0 (0)
Decreased platelet count	4 (9.6)	1 (2.4)
Vomiting	3 (7.3)	0 (0)
Hypothyroidism	1 (2.4)	0 (0)
Elevated aspartate aminotransferase	3 (7.3)	0 (0)
Increased blood bilirubin	2 (4.9)	1 (2.4)
Constipation	1 (2.4)	0 (0)

### Treatment Response Based on CA19-9 and Gene Mutations

Of the 41 patients, 48.8% (n = 20) showed decreased CA19-9 concentrations after initiation of lenvatinib. Prediction of DCR using CA19-9 had a sensitivity of 59%, but specificity was substantially higher at 88.8% (**Table 2**).

**Figure 4:**
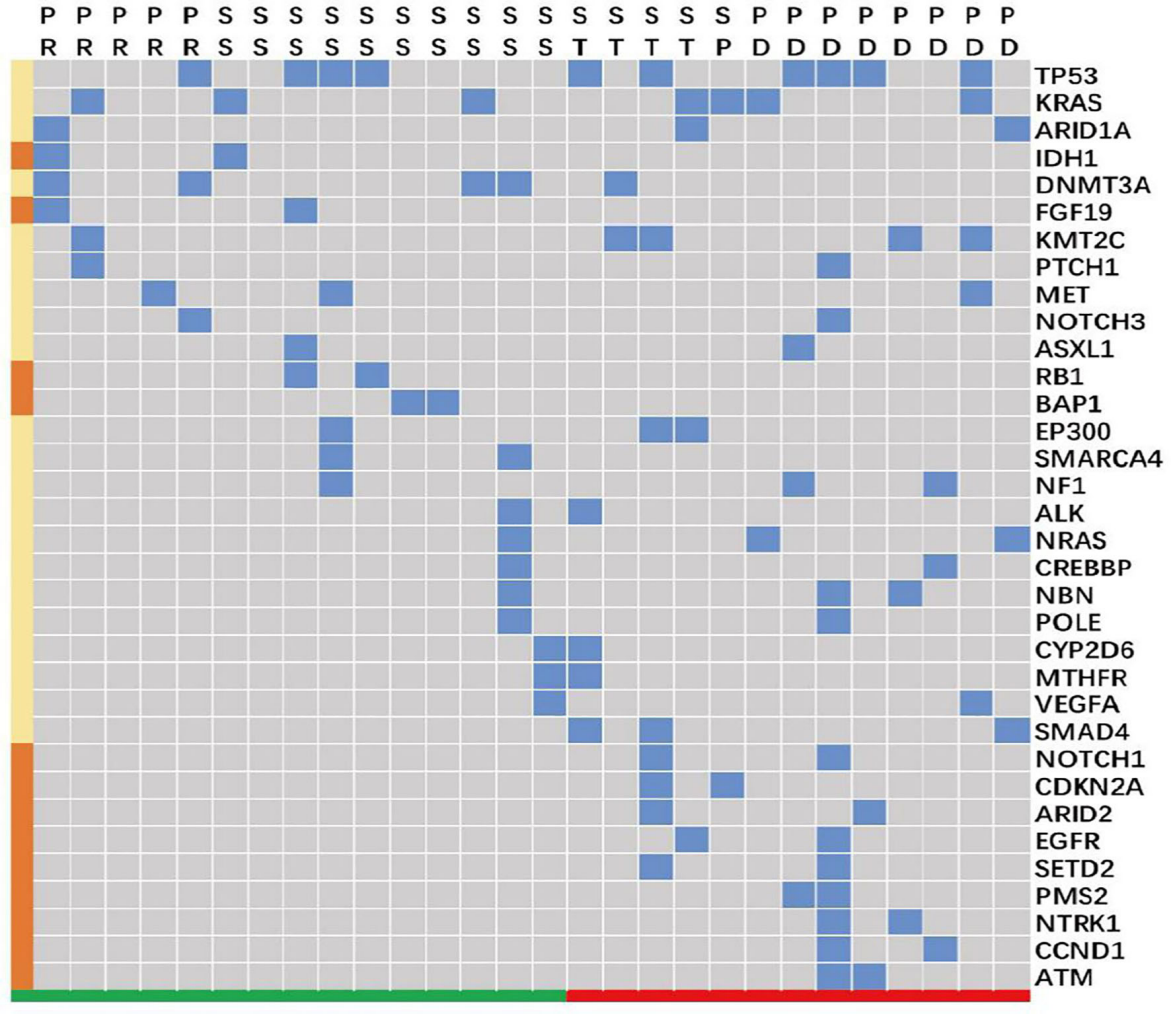
Gene alterations in patients with biliary tract carcinoma (n = 28). SS and PR (colored in green along the bottom) clustered as a group characterized by tumor size reduction in response to treatment. ST, SP, and PD (colored in red) clustered as a ‘non-response’ group, which included patients who did not respond to treatment with tumor size reduction. The blue blocks represent the presence of an alteration in the indicated gene across individual patients, and the brown blocks along the left indicate that the gene alteration was present only either in the tumor size reduction group (*IDH1*, *FGF19*, *RB1*, and *BAP1*) or in the “unshrinking” group (*NOTCH1*, *CDKN2A*, *ARID2*, EGFR, *SETD2*, *PMS2*, *NTRK1*, *CCND1*, and *ATM*). PD, progressive disease; PR, partial response; SS, diminished tumor size that did not reach the partial response standard; ST, a stable disease without any significant tumor size change.

Gene mutation data from 28 patients in the BTC group was analyzed. These patients were divided into two groups by tumor response: a group with reduced tumor size (n = 15) and a group characterized by stable or enlarged tumors (the ‘non-response’ group, n = 13). The majority of BTC mutations involved *TP53* (n = 10), *KRAS* (n = 7), and *DNMT3A* (n = 5). Alterations in *IDH1*, *FGF19*, *RB1*, and *BAP1* appeared in patients with tumor size reduction, but not in those with non-responding tumors. On the other hand, alterations in *NOTCH1*, *CDKN2A*, *ARID2*, *EGFR*, *SETD2*, *PMS2*, *NTRK1*, *CCND1*, and *ATM* were found in patients with non-responding tumors, but not in those with tumor size reductions (**Figure 4**).

## Discussion

The purpose of this study was to evaluate the safety and efficacy of lenvatinib as beyond first-line treatment in patients with unresectable BTC. Lenvatinib showed antitumor activity, with an ORR of 12.2% (95% CI: 1.7–22.7), a DCR of 78.0% (95% CI: 64.8%–91.3%), and a median OS of 11.4 months (95% CI: 6.6–16.2). A majority of the enrolled patients (95.1%, 39/41) experienced ≥1 treatment-related AE, and the incidence of grade 3–4 AEs was 19.5% (n = 8). The most common AE was fatigue 41.3% (n = 19), followed by hypertension 39.1% (n = 18). The sensitivity and specificity of CA19-9 serum concentration before and after treatment for predicting tumor shrinkage were 77.7% and 73.9%, respectively, while the sensitivity and specificity for predicting DCR were 59.3% and 88.8%. The most commonly mutated genes in patients with tumor shrinkage were *DNMT3A*, *TP53*, and *KRAS*.

Surgical resection is the best option for patients with localized disease. However, most BTC patients are at an advanced stage when diagnosed (5). The ABC-02 trial established the combination of GC as a first-line treatment in BTC patients, with GC showing a better OS than gemcitabine alone (median OS: 11.7 months *vs* 8.1 months). Only around 26% of patients respond to this chemotherapy regimen, and the efficacy is limited (3). For patients with disease progression following treatment with cisplatin and gemcitabine, the FOLFOX as a second-line treatment showed a modest survival benefit from the ABC-06 trial (6). The study reported an mOS of 6.2 months, mPFS of 4.0 months in the ASC plus mFOLFOX arm. However, ORR was only 5% and DCR was 33%. For patients who progress after the above therapies, the subsequent treatment options are limited. Moreover, some patients have difficulty tolerating the many side effects of chemotherapy drugs. Therefore, additional therapies are needed.

Multi-kinase small molecule inhibitors have been investigated the efficacy and safety in patients with biliary tract cancers (BTCs). A phase II study of sorafenib, a kinase inhibitor of VEGFR2/3, PEGFR, and RAF, evaluating the safety and efficacy of sorafenib in patients with unresectable or metastatic gallbladder carcinoma and cholangiocarcinoma was terminated because it did not result in objective responses (ORR:0%) (18). Regorafenib as a single agent in patients with advanced BTC showed clinical efficacy with an ORR of 11% and an mPFS of 15.6 weeks (90% CI: 12.9–24.7 weeks) (19). The activity of anti-VEGF targeted therapies as monotherapy in advanced BTC is not very satisfactory. Recently, infigratinib (BGJ398), a selective FGFR kinase inhibitor, has shown promising results against chemotherapy-refractory CCA harboring FGFR2 fusions (NCT02150967). In that study, the ORR was 14.8% (18.8% in those with *FGFR2* fusions), the DCR was 75.4% (83.3% in those with *FGFR2* fusions), an estimated median PFS was 5.8 months (95% CI: 4.3–7.6) (20). The FDA has approved the infigratinib for patients with locally advanced or metastatic cholangiocarcinoma. Another study showed that ivosidenib (AG-120) targeted *IDH1* mutations could improve survival outcomes in participants with histologically confirmed *IDH1*-positive chemotherapy-refractory cholangiocarcinoma, with a PFS of 2.7 months (IQR 1.6-4.2) (21). Ivosidenib and infigratinib are effective for patients with specific gene mutations. Our study enrolled patients regardless of specific gene mutation.

Lenvatinib not only inhibits VEGFR1-3, FGFR1-4, PDGFRa, KIT, and RET but also demonstrates immunomodulatory activity. Lenvatinib has proven to be effective in many types of cancer. However, there have been few studies on the efficacy of lenvatinib in BTC. A phase II study of lenvatinib monotherapy as second-line treatment in 26 patients with unresectable BTC reported an ORR of 11.5%, a median PFS of 3.19 months, and a median OS of 7.35 months (Study 215). The present study showed better results than Study 215, with a median PFS of 3.8 months and an OS of 11.4 months (22). An important factor behind the longer OS and PFS is that the BTC subtypes were represented differently between studies; patients with ICC accounted for only 23.1% of patients in that study compared to 82.9% in our study. Moreover, all patients in that study received a higher dose of lenvatinib (24 mg orally once daily), in contrast to our study in which the lenvatinib dosage was individualized based on the patient’s weight, to a maximum dose of 12 mg daily.

Evidence has shown that lenvatinib exerted immunomodulatory effects (23, 24). We have reported that lenvatinib plus pembrolizumab as a non-first-line treatment for BTC was potentially effective and tolerable, with an ORR of 25%, a CBR of 40.5%, a median PFS of 4.9 months, and a median OS of 11.0 months (17). These results showed that lenvatinib plus anti-PD-1 might have promising antitumor activity in patients with advanced BTC. Dual anti-PD-1 and VEGF/VEGFR blockade may reprogram the tumor microenvironment and enhance the antitumor efficacy of immunotherapy targeting PD1/PDL1 (25).

Regarding safety and tolerability, we found that 95.1% of patients experienced treatment-related AEs; however, the incidence of grade 3–4 AEs was 19.5%, and there were no grade 5 AEs. Of the grade 3–4 AEs, diarrhea and hypertension were the most common (4.9% each). The most common any-grade AE was fatigue (41.3%, n = 19), followed by hypertension (39.1%, n = 18), and these side effects were generally tolerable. In Study 215, 80.8% of patients experienced grade ≥3 treatment-emergent AEs, which is over four times the rate observed in our study (22). In our study, patients with a bodyweight <60 kg received 8 mg of lenvatinib daily, and those with a bodyweight ≥60 kg received 12 mg. Thus, lenvatinib side effects appear to be closely related to the administered dose, and this may explain the differences between the safety findings of the two studies. Besides, we timely found and treated the AEs of each patient through social platforms. As hypertension, diarrhea, and proteinuria are serious threats to a patient’s quality of life, it is important to ensure timely control of AEs and interrupt lenvatinib treatment if necessary.

Tumor biomarkers represent tumor activity under certain circumstances, and clinicians may use changes in tumor biomarkers to assist in understanding and predicting treatment effects. CA19-9 levels play an important role in the diagnosis of BTC. Results from this study suggest that a downward trend of CA19-9 levels after initiating lenvatinib is representative of response to treatment. Upon further analysis, we found this response to be highly sensitive and specific for predicting the reduction of tumor volume. Therefore, CA 19-9 may serve as a biomarker in the treatment of BTC, and clinicians may be able to adjust lenvatinib treatment plans in accordance with decreasing serum markers. However, this is an area that requires further validation in future studies.

BTC is a highly heterogeneous malignancy (26). Gene sequencing is necessary to pave the way for new treatment options and personalized medicine. In a study of BTC targets, potential driver mutations were detected in a majority of patients, and 40% of these mutations could be targeted with therapeutics (27). The frequency of gene alterations in BTC is comparatively high, particularly so in *IDH* as well as in the *TP53*, *KRAS*, and *FGFR* pathways (28). Additionally, the three subtypes of BTC have substantial molecular heterogeneity (29). The FGFR fusions, TP53, KRAS, IDH1/2, and BAP1 mutations, are the most common mutation in iCCA, while PRKACA, PRKACB fusions, and ELF3 mutations, frequently occur in p/dCCA (29, 30). Loss-of-function mutations of tumor suppressor genes have been associated with a worse prognosis in BTC. Interestingly, Apurva Jain and colleagues found that FGFR genetic aberrations may have a positive impact on OS in younger patients with an indolent disease course (31). Identification of distinct patient subgroups is associated with targeted therapies. Indeed, gene sequencing leads the way for precision medicine in BTC (32). Besides, another study found BRCA-associated protein 1(BAP1) mutation in CCA may be associated with aggressive disease and poor response to standard therapies (33).

This study had several limitations. Firstly, the study evaluated the anti-tumor activity of lenvatinib as a non-first-line therapy in patients with advanced BTC using a single-arm design and with a limited number of patients, which limits the strength of evidence and lacks a comparator. Secondly, patients enrolled are from diverse regions of China, which may lead to the bias of the heterogeneity caused by geography. In addition, this study is an investigator-initiated study with underlying selection bias and participant bias. Finally, the mPFS in this study was lower compared to FOLFOX chemotherapy.

In conclusion, the results of this study suggest that lenvatinib which was individualized based on the patient’s weight has promising clinical activity against advanced BTC. Besides, AEs caused by levatinib appear to be closely related to the administered dose, and timely detection and treatment of AEs can reduce the occurrence of some AEs. Changes in tumor biomarkers before and after administration of lenvatinib can potentially be used to predict changes in the size of tumors during therapy, with a sensitivity and specificity of approximately 70%. Gene detection is likely to provide guideposts for precision medicine as we move forward in identifying effective therapies for BTC.

## Data Availability Statement

The original contributions presented in the study are included in the article/supplementary material. Further inquiries can be directed to the corresponding authors.

## Ethics Statement

The studies involving human participants were reviewed and approved by Institutional Review Board and Ethics Committee at Peking Union Medical College Hospital. The patients/participants provided their written informed consent to participate in this study.

## Author Contributions

YCW, DXW, and XBY collected the data and wrote the manuscript. XY, YYW, JYL, JXZ and ZHL helped collect literature and participated in discussions. MG and HZ designed and verified the study. YLM and XTS examined the language. All authors contributed to the article and approved the submitted version.

## Funding

This work was supported by the International Science and Technology Cooperation Projects (2016YFE0107100), the Capital Special Research Project for Health Development (2014-2-4012), the Beijing Natural Science Foundation (L172055 and 7192158), the National Ten-thousand Talent Program, the Fundamental Research Funds for the Central Universities (3332018032), and the CAMS Innovation Fund for Medical Science (CIFMS) (2017-I2M-4-003 and 2018-I2M-3-001).

## Conflict of Interest

The authors declare that the research was conducted in the absence of any commercial or financial relationships that could be construed as a potential conflict of interest.

## Publisher’s Note

All claims expressed in this article are solely those of the authors and do not necessarily represent those of their affiliated organizations, or those of the publisher, the editors and the reviewers. Any product that may be evaluated in this article, or claim that may be made by its manufacturer, is not guaranteed or endorsed by the publisher.
